# Major Depressive Disorder, Inflammation, and Nutrition: A Tricky Pattern?

**DOI:** 10.3390/nu15153438

**Published:** 2023-08-03

**Authors:** Veronique Bernier, Marie-Hélène Debarge, Matthieu Hein, Sarah Ammendola, Anais Mungo, Gwenole Loas

**Affiliations:** 1 Department of Psychiatry and Sleep Laboratory, Erasme Hospital, Université Libre de Bruxelles—ULB, 1070 Brussels, Belgiummatthieu.hein@hubruxelles.be (M.H.); gwenole.loas@hubruxelles.be (G.L.); 2 Department of Psychiatry, Brugmann University Hospital, Université Libre de Bruxelles—ULB, 1020 Brussels, Belgium

**Keywords:** major depressive disorder, inflammation, nutrition, Western diet

## Abstract

Major depressive disorder (MDD) is a psychiatric disease associated with inflammation. The Western diet (WD) is a high-fat, high-sugar diet also associated with inflammation. We aimed to show whether the diet of MDD patients was a WD and could act as a risk factor in this context. We conducted a transversal study of MDD patients and controls (CTRLs) without comorbidities. We performed blood analyses including C-reactive protein (CRP), a diet anamnesis, and an advanced glycation end-product assessment. We found that 34.37% of MDD patients had a CRP level above 3 to 10 mg/L, which remained higher than CTRLs after adjustments (sex, BMI, age, smoking status). The MDD patients had an excess of sugar and saturated and trans fatty acids; a deficiency in *n*-3 polyunsaturated fatty acid, monounsaturated acid, dietary fibers, and antioxidants; a high glycemic load; and aggravating nutritional factors when compared to the CTRLs. We found correlations between nutritional factors and CRP in univariate/multivariate analysis models. Thus, MDD patients showed an elevated CRP level and a WD pattern that could contribute to sustaining an inflammatory state. Further studies are required to confirm this, but the results highlighted the importance of nutrition in the context of MDD.

## 1. Introduction

### 1.1. Depression and Inflammation

The burden of depression is high, affecting about 300 million people worldwide, with the majority being women, young people, and the elderly [1]. Furthermore, depression is now the leading cause of disability in the world [2]. 

Depression is a psychiatric pathology associated with inflammation [3,4]. This is a systemic inflammation defined by a C-reactive protein (CRP) level that is above 1 to 10 mg/L [5]. The 2019 meta-analysis conducted by Cambridge University determined that 27% of depressed patients have a CRP level above 3 to 10 mg/L, and 58% above 1 to 10 mg/L [5]. The CRP level range between 3 and 10 mg/L is referred to as low-grade inflammation (LGI). LGI has already been identified as a risk factor for cardiovascular disease [6]. 

According to the Diagnosis Statistic Manual of Disorder (DSMV), major depressive disorder (MDD) is defined by a minimum of five out of nine of the following criteria: anhedonia, depressive mood, weight variation, sleep disturbance, an alteration in psychomotor activity, tiredness, feelings of guilt or worthlessness, suicide attempts, and cognitive disturbance. One of the first two criteria at minimum is required. These criteria are qualitative but also quantitative, as they must possess characteristics of frequency and duration. Due to the heterogeneity of assessments, the worldwide prevalence of MDD is difficult to measure accurately and has been evaluated at about 4.7% [7]. In 2021, for Europe, the prevalence of major depressive episodes was estimated at about 6.38%, with a higher rate for women than men [8].

The main theory that underlines the link between inflammation and depression is the sickness behavior [9]. During an infectious or inflammatory disease, the cytokines and the immune cells could reach the central nervous system (SNC) through the more permissive regions of the blood–brain barrier (BBB), such as the circumventricular organs and the choroid plexus [10]. There is also a neural pathway involved via the nerve afferent fibers [11]. Both of these signals are responsible for the somatic symptoms of inflammation: fever, tiredness, anorexia, and sleep disturbance. They are also responsible for the psychological symptoms of inflammation: social withdrawal, anhedonia, anxiety, and a depressive mood. When the infection ends, the symptoms stop. These symptoms match behavioral changes such as withdrawal into oneself. These types of changes could be interpreted as a positive adaptation of immunity that occurred during the process of evolution. The prehistoric human needed to adopt a “save energy behavior” in order to recover when infection occurred [12]. 

Clinical evidence of the sickness behavioral theory has been found in hepatitis C cytokine treatment. Gamma interferon treatment leads to symptoms of depression for about 40% of patients [13]. Interestingly, these patients presented higher psychological and genetic fragility as assessed before treatment [13]. These psychological symptoms were improved by a conventional antidepressant pre-treatment [14].

There is an overlap between the psychological symptoms of inflammation and those of depression, including social withdrawal, a depressive mood, anhedonia, and anxiety. The main difference with an infectious or inflammatory disease is that depression is a chronic disease. Thus, the inflammatory context of depression would continue to act on the brain and on these symptoms. Therefore, inflammation could contribute to sustaining depressive symptoms and could act as a risk factor. The disruption of the BBB identified in MDD patients and the depressive-type behavior in animal models could reinforce the passage of the immune cells and cytokines into the brain [15,16,17].

There is also an interesting alternative theory about the link between depression and inflammation that is related to treatment resistance (TR). TR is an important issue in psychiatry and, in particular, for depression [18]. Moreover, TR is a particularly common severity criterion of MDD. The etiology of TR is diverse; first of all, it is related to matching a good diagnosis with the appropriate drug in the right dose [18]. However, an elevated CRP level is associated with treatment resistance in depressed patients [19,20,21]. Inflammation is also associated with a bad response to antidepressants [22]. Certain studies have investigated immunotherapy and found that it could be effective in the case of depressive patients with peripheral systemic low-grade inflammation [23]. Others have investigated the efficiency of the type of depressor related to the CRP level [24]. An interesting clinical study assessed two treatment strategies with two types of anti-depressants: a selective serotonin reuptake inhibitor (SSRI) and an SSRI associated with a selective catecholamine reuptake inhibitor (SCRI). The study results showed that the most effective monotherapy was SSRIs for a CRP level < 1 mg/L. Conversely, the treatment response for the SSRI + SCRI was better for a CRP level > 1 mg/L [25]. 

The depletion of serotonin has also been extensively suspected of promoting depression. Tryptophane is the precursor of serotonin. The idolamine/kynurenine pathway is a way of catabolizing tryptophane and is triggered by pro-inflammatory cytokines. In the context of SCI, the activation of the kynurenine pathway could lead to the release of toxic metabolites, decreasing the amount of tryptophane and, as a result, the anabolism of serotonin [10,26]. 

There is not yet a consensus about the role of immunity in terms of the neurobiological cause of depression. Furthermore, inflammation could act at the same time as other neurobiological factors [18]. More studies are required, but as we noticed previously, several studies are converging consistently in this direction.

From an epidemiologic point of view, there is a high prevalence of diabetes and obesity in depression. Obesity is a risk factor for depression, and a diabetic person is two times more likely to suffer from depression [27,28]. These pathologies are also associated with inflammation [29,30,31]. In this inflammatory context, it may be difficult to separate the role of comorbidities such as obesity and diabetes from depression “per se”. Therefore, in order to avoid potential biases, the first objective of this study was to verify whether MDD patients without comorbidities had higher CRP levels than the CTRLs. 

### 1.2. The Western Diet, a Risk Factor in the Context of Depression? 

In the West, the prevalence of metabolic diseases and depression have increased at the same time as a profound shift has occurred in human lifestyles (i.e., urbanization, sedentariness, and nutrition) [32,33,34]. The new dietary pattern, the so-called Western diet (WD), is a high-fat, high-sugar diet with an excess of nutrients such as saturated fatty acids (SFAs); trans fatty acids (TFAs); and a deficiency in protective nutrients such as *n*-3 polyunsaturated fatty acids (PUFAs), monounsaturated fatty acids (MUFAs), dietary fibers (DFs), antioxidants (AOs), and minerals. *n*-6 PUFAs and *n*-3 PUFAs are also known as omega 6 and omega 3. The WD is rich in meat, animal fats, palm oil, ultra-processed foods, refined carbohydrates, free sugar (mono- and disaccharides), and salt. Furthermore, it is low in vegetables, fruits, whole grains, nuts, legumes, olive oil, and fish [35,36]. 

The WD is rich in foods that are able to increase glycemia dramatically after ingestion and absorption (e.g., sugar, processed food, and refined carbohydrates) [34,37]. This ability to trigger postprandial hyperglycemia in a physiological range is an important issue with the WD and emphasizes its obesogenic nature [34,38]. This ability is multifactorial and (mainly) due to the carbohydrate (CHO) content (e.g., starch and sugar), but it also due to the method of processing the food (preparation and cooking), as well as the dietary fiber content and the presence of other macronutrients (e.g., fat). The food glycemia increase is assessed by the glycemic index (GI). The GI is the capacity of a food to increase glycemia after ingestion, compared to glucose. The glycemic load (GL) assesses the glycemic impact of the serving by taking into account the quantity of CHO consumed [39]. Due to its high content of added sugar, processed food, and refined CHO, the WD has a high glycemic load pattern [34,37].

The WD is also associated with a high level of advanced glycation end products (AGEs). The AGEs are a large and heterogenous family of glycotoxins. They are all formed by a reaction between a reduced sugar and a free amino acid function (i.e., the Maillard reaction). The nature of AGEs can be endogenous or exogenous. The exogenous form comes mainly from the diet and cigarette smoke. The dietary AGEs occur due to a hot, dry cooking process of meats, fat, cheese, and nuts and, in lower proportions, in deep-cooked or fried CHO (e.g., crips and crackers) [40]. The WD involves dry and hot cooking processes that are rich in meat, animal fats, and sugar [40,41,42]. Therefore, the WD is associated with a high level of AGEs, and an elevated AGE level has also been associated with depression [41,43]. 

Overall, the WD is associated with inflammation and with the main pathologies that affect the West: cardiovascular disease, diabetes, obesity, and (certain) cancers [36,41,44,45,46]. The links between the WD and inflammation have been closely studied and are numerous. From a nutritional point of view, the WD features a poor balance between pro-inflammatory and anti-inflammatory nutrients. The pro-inflammatory nutrients are SFAs (e.g., palmitate) and industrial TFAs (when they are in excess). In the context of a high-fat diet like the WD, the intracellular accumulation of palmitate could lead to the inhibition of the AMP-activated protein kinase and then to the production of reactive oxygen species (ROS) in the mitochondria. The ROS could in turn activate the inflammasome NLRP3 (NOD-LRR and the pyrin-domain-containing protein 3) and the release of pro-inflammatory cytokines such as interleukin (IL)1β and IL-18 [47]. Industrial TFA comes mainly from partially hydrogenated vegetable oil, the prolonged deep frying of food, and the deodorization of marine oil. TFAs are associated with the activation of the pro-inflammatory nuclear factor-kappa B (NFκB), as well and an increase in the CRP level [48,49].

The protective and anti-inflammatory nutrients are *n*-3 PUFA, MUFA, AO, DF, and magnesium [35,40]. The *n*-3 PUFA alpha linolenic fatty acid (ALA) and the *n*-6 PUFA linoleic fatty acid (LA) are both essential polyunsaturated fatty acids. They are precursors of arachidonic acid (AA) in the case of LA and of eicosapentaenoic acid (EPA) and docosahexaenoic acid (DHA) in the case of ALA. DHA and EPA are also the main components of the *n*-3 PUFA fish oil [50]. AA is the precursor of the pro-inflammatory proteins leukotriene (LK) and prostaglandine (PG). Conversely, EPA and DHA give rise to metabolites that have lower inflammatory or anti-inflammatory properties. EPA and DHA are the precursor of anti-inflammatory metabolites that are the mediators of the final state of inflammation: maresine (MA), protectine (PO), and resolvine (RE). The ratio of *n*-6 PUFA/*n*-3 PUFA is important to take into account, because they use the same enzymatic pool to synthesize their metabolites along the whole metabolic chain. Therefore, an increase in ALA, DHA, or EPA leads to a decrease in AA, LK, or PG for the benefit of the anti-inflammatory eicosanoids MA, PO, and RE, respectively [51,52]. 

The mechanism underlying the DHA/EPA inflammatory properties is their incorporation in the cell membrane and the capacity of free *n*-3 PUFA to bind to cellular receptors [50]. The context of a rich EPA/DHA diet enhances the EPA/DHA proportions in the cell membrane. This increases the membrane fluidity, leading to the disruption of the signaling platform (lipid rafts) and the blunting of the Toll-like receptor (TLR) signal and the NFkB pro-inflammatory pathway [53]. Furthermore, free ALA, EPA, and DHA can bind the G-protein-coupled receptor (120 GPR). This receptor activation leads to an inhibition in the phosphorylation of the transforming growth factor beta-activated kinase 1 protein (TAK1) and then both the TLR and TNF alpha pro-inflammatory pathways [50,54]. EPA and DHA could also activate the anti-inflammatory transcriptor factor peroxisome proliferator-activated receptor gamma (PPAR-γ) [50]. The modulation of the pro-inflammatory receptors/transcriptor factors leads to a downsizing in the expression of pro-inflammatory cytokines [50,54]. 

Interestingly, the dietary intake of *n*-3 PUFA in the interferon treatment of hepatitis C seems to improve the psychological symptoms of inflammation [55]. Moreover, the administration of EPA/DHA and a conventional antidepressant could contribute to reversing the increase in kynurenine and cytotoxic metabolites induced due to the activation of the kynurenine pathway by pro-inflammatory IL-1β [56]. IL-1β is increased in depressed patients and could be involved in the sickness behavior [57,58].

Monounsaturated fatty acids (MUFAs) could promote the oxidation of SFAs and facilitate their incorporation into triglycerides [59,60]. They have been shown to limit reticulum endoplasmic oxidative stress due to the palmitate in the macrophage [61]. The substitution of SFAs by MUFAs diminishes the activation of the inflammasome NLRP3 [62].

Antioxidants (AOs) such as vitamins A, E, and C, as well as polyphenols, are key actors in reducing the pro-inflammatory process [63,64]. They have the properties to offset free radicals and to enhance the AO endogen pool. Free radicals are normal metabolite products, but their accumulation could lead to oxidative stress (OS). OS could, in turn, lead to DNA and mitochondrial damage and could trigger the inflammatory pathway. Moreover, an increased OS level is associated with major depression [65]. The main source of AOs are fruits, vegetables, and spices [64]. Polyphenols act in multiple ways as inhibitors of the pro-inflammatory pathway (e.g., NFκB) or as modulators of TLR activity and the production of pro-inflammatory proteins. They inhibit the production of AA enzymes, leading to a decrease in its LK and PG metabolites as a result. They also inhibit the enzymes involved in the production of ROS. Interestingly, curcumin could upregulate the endogenous AO superoxide dismutase (SOD), as well as catalase and glutathione peroxidase (GSH-Px) [66]. Vitamins C and E are associated with a decrease in pro-inflammatory cytokines and the CRP level [66,67,68]. Finally, the activity of olive oil phenolic compounds has been widely studied, and its efficiency has been shown against reactive oxygen species [69]. Polyphenols cannot be assessed by a nutritional database, but the dietary assessment of the main food sources (fruits and vegetables) allows us to estimate the food intake [64].

The dietary intake of magnesium is inversely associated with oxidative stress and CRP [70,71]. The biomechanism has not yet been defined, but in animal models it has been shown that a depletion of magnesium could trigger the activation of macrophages and the release of pro-inflammatory cytokines [70]. A deficiency in magnesium is associated with depression [65,72,73]. Zinc also seems to be involved in the modulation of inflammation and oxidative stress [41,65,74]. A chronic zinc deficiency is associated with an increase in pro-inflammatory cytokines [74]. A deficiency in magnesium and zinc is associated with depression as well [65,72,73].

Dietary fibers (DFs) are the main substrate of the gut microbiota. A diet rich in dietary fibers enhances the diversity and the number of the microbiota species. This abundance is considered protective for an effective gut functional barrier. The fibers are fermented by the bacteria, which in turn release short-chain fatty acids (SCFAs): butyrate, acetate, and propionate. SCFAs such as butyrate have trophic (colonocyte) and anti-inflammatory properties. The dietary fibers and the SCFA microbiota producers also maintain a good balance in the gut mucus layer’s thickness. This is also an important issue for preserving the integrity of the gut barrier. High-fiber diets and SCFAs are associated with an increase in the quantity of antimicrobial peptides and an upregulation in the tight junctions [75].The fermented fibers can also release compounds such as ferulic acid. This metabolite also has anti-inflammatory and antioxidant properties [75].

Dietary fibers are inversely associated with the CRP level [76]. In the WD, the lack of dietary fibers shapes the gut microbiota into species that can metabolize the available substrates, mainly meat and inner mucus [77,78,79]. Conversely to carbohydrates’ fiber fermentation, the metabolization of amino acids releases cytotoxic and pro-inflammatory metabolites and leads to a decrease in the antimicrobial properties. The WD is associated with a decrease in both the number and the diversity of the microbiota species [80]. Thus, the WD could weaken the gut functional barrier and could promote inflammation [75].

The nature of carbohydrates is an important issue in mental health [37,38]. The WD is rich in added free sugars (FSs), which are mono- and disaccharides (e.g., glucose, fructose, and sucrose), and refined CHO; both have a high GI. The release of insulin due to a high glycemic load (HGL) can lower the glycaemia to a threshold that could trigger counter-regulatory hormones and could lead to mood disorders such as anxiety and irritability [81]. Regarding the purpose of our study, a HGL diet in healthy subjects is associated with postprandial glycaemia but also oxidative stress and the activation of the pro-inflammatory NFKB signaling pathway [46,82]. A high GI and GL are also associated with biomarkers of inflammation such as CRP [41,83,84]. Conversely, it has been shown that a low GL diet reduces the CRP level [85,86]. A healthy population with a high glycemic diet has also been associated with higher depressive symptoms [87]. The association between a diet rich in fat and sugar and poor mental health has also been shown in a large cohort study [38,88]. 

As we have seen previously, the WD has a high level of AGEs due to its food and cooking patterns. Endogenous AGEs are normal byproducts of metabolism. Both exogenous and endogenous AGEs have to be counteracted by detoxification mechanisms. If not, their accumulation could be toxic, impairing cell function and sustaining oxidative stress and inflammatory contexts by bending their intracellular receptor, RAGE [89]. Dietary AOs, mainly polyphenol, could prevent the formation of AGEs and block the RAGE receptor [90,91]. The WD is structurally poor in protective nutrients such as AOs, which could offset oxidative stress. Thus, the WD is extensively associated with oxidative stress and inflammation. 

Whereas the first objective of this study was to show that MDD patients without comorbidities had an elevated CRP level compared to the CTRLs, the second goal was to assess whether their diet, through the balance of the pro-inflammatory (SFAs, TFAs, *n*-6 PUFA, and sugars) and anti-inflammatory nutrients (AGMI, *n*-3 PUFA, DFs, and Aos), but also the glycemic load and the AGE level, could act as a risk factor. We aimed to identify the pro-inflammatory global feature of MDD patients’ diets. Whereas the CTRL population was also presumed to have a WD, we aimed to show that the diet of MDD patients presented more nutritional risk factors than that of the CTRLs. Finally, to confirm the link between nutrition and inflammation in this context, we performed correlation tests between the CRP levels and the nutritional risk factors in the uni- and multivariate analyses. 

## 2. Materials and Methods

### 2.1. Population

We conducted a transversal study with MDD inpatients and controls. The patients were hospitalized in the psychiatric department of the Hôpital Erasme, Cliniques Universitaires de Bruxelles and the Centre Hospitalier Universitaire Brugmann. The inclusion criteria were as follows: male or female; aged over 18 and below 75; and with no comorbidities such as diabetes, obesity, cancer, or inflammatory disease. The definition of obesity was based on the body mass index (BMI), which is the ratio of the weight (kilograms) to the square of the height (meters). Obesity starts with a BMI ≥ 30. Pregnancy, addiction, and eating disorders were excluded, as well as other psychiatric diseases. A CRP level > 10 mg/L is suspicious and presumed to be due to infection or a disease. In order to avoid any potential biases, we excluded a CRP level higher than this threshold. The control population adhered to the same criteria with no psychiatric antecedents. Forty-one inpatients and thirty-eight controls were assessed. Thirty-two inpatients and thirty-one CTRLs matched the inclusion criteria.

All subjects gave their informed consent for inclusion before they participated in the study. The study was conducted in accordance with the guidelines in the Declaration of Helsinki, and the protocol was approved by the Ethics Committee of Erasme Hospital (P2020/409) on 9 November 2020. 

### 2.2. Method

After a psychiatric assessment based on DSM V, a blood analysis was performed that included the CRP level, total cholesterol, low-density lipoprotein cholesterol, high-density lipoprotein cholesterol, triglycerides, glycemia, glycated hemoglobin, and liver and kidney functions. A dietician performed the diet anamnesis, which was based on the alimentary history interview technique and a food frequency questionnaire [92,93]. The multiple-pass recall method was used to improve the reliability of the information [94]. The AGEs were measured with the AGE skin reader, a non-invasive technique of immunofluorescence (see Materials subsection) [95]. The protocol was the same for both populations.

In order to calculate the nutrient intake, we used Nubel Pro database software [96]. Concerning the free sugar intake (mono- and disaccharides), the Nubel database could not separately calculate the added and natural sugars. Thus, we used the EFSA recommendation of 20% of the maximum sugar daily intake, taking into account that the fruit intake (the main natural sugar source) should be below the nutritional reference values [97]. The omega 3 level reported by Nubel nutritional software is the overall intake of *n*-3 PUFAs: ALA, EPA, and DHA. The TFA level reported in the Nubel table is also the overall intake of natural and industrial TFAs. For the glycemic index, we used the Foster–Powell table [98]. The glycemic load was calculated by multiplying the carbohydrate quantity of a food by the glycemic index and dividing by one hundred [39]. 

In order to estimate the total theorical daily energy expenditure (TDEE), we first calculated the basal metabolite rate (BMR), which was based on the Henry equation [99]. We multiplied the BMR by the physical expenditure coefficient (PAL) that was determined during diet anamnesis [100]. We calculated the gap between the daily caloric intake and the TDEE. For 18.5 < BMI > 25, we used a corrected weight, which was calculated with the BMI equation and the closer value of the standard range. 

### 2.3. Materials

The CRP blood assessments were performed, as with the rest of the blood analyses, in the Brussels University Laboratory (LHUB-ULB). The CRP was measured (in plasma) using a Roche CRP4_Cobas with a minimum detection level of 0.3 mg/L. The nutritional database was the NUBEL PRO food planner application [96]. The nutritional references (NR) were based on the nutritional and food recommendations of the Superior Health Council of Belgium 2016–2019 [100,101].

The AGE assessment was performed using an AGE skin reader. AGEs exhibit autofluorescence properties and can accumulate in the skin. Therefore, using an excitation light source of 300–420 nm, the fluorescence of the skin can be measured on the arm of a patient to assess the AGEs. This test can be performed only with non-pigmented skin [95].

### 2.4. Biomarkers

The CRP level is a relevant biomarker of peripheral systemic low-grade inflammation [4,102,103]. In healthy people, a normal CRP level is: ≤0.55 mg/L for a man and ≤1 mg/L for a woman (104). The systemic CRP level could be influenced by age, weight, sex, and smoking status. 

As CRP is the main biomarker used for both the WD and depression, we also utilized it herein [5,21]. However, we took into account the influencing parameters seen above in our statistical calculations.

### 2.5. Statistics

The statistical calculations were performed with SPSS 29.0, Jamovie 1.6.5, and Jasp software. For the CRP comparison level, we used non-parametric Mann–Whitney tests and the quantile and logistic regression test for adjustment. For the nutritional calculation, the data are presented as the mean ± the standard deviation of the mean for each nutrient and food category. We used, depending on the distribution normality, unilateral or bilateral parametric tests (mainly Student’s test) and non-parametric tests (Mann–Whitney and Wilcoxon). We completed the univariate analysis between the nutritional factors and CRP level with the Spearman coefficient of regression, and we conducted a principal component analysis (PCA)/Varmix model for the multivariate analysis. In all tests, a *p* value < 0.05 was considered statistically significant. 

## 3. Results 

### 3.1. CRP Data 

**Figure 1:**
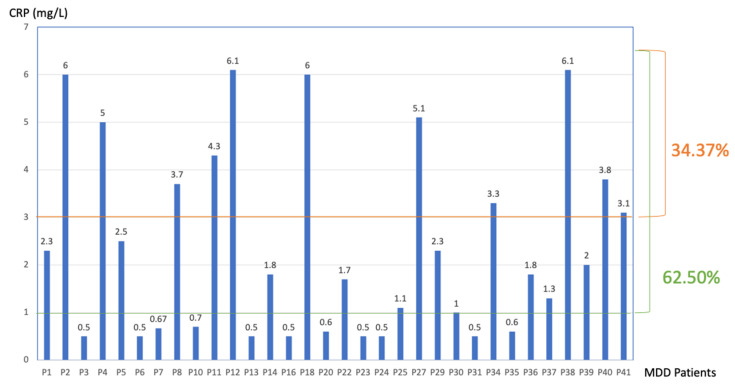
The CRP level (mg/L) of the MDD patients. The data are presented as follows: p(n) = patient (n). The first threshold was for a CRP > 1 mg/L (the first longitudinal line), the second was for a CRP > 3 mg/L, which is the range of low-grade inflammation (the higher longitudinal line). The figure of 34.37 is the percentage of patients in the range above 3 to 10 mg/L, and the figure of 62.50 is the percentage of patients that had a CRP level above 1 to 10 mg/L.

A total of 62.50% of the MDD patients had a CRP above 1 but below 10 mg/L. A total of 34.37% of the MDD patients had a CRP above 3 but below 10 mg/L (Figure 1). The MDD CRP level was higher than in the control population, *p* = 0.019 (Table 1). 

**Figure 2:**
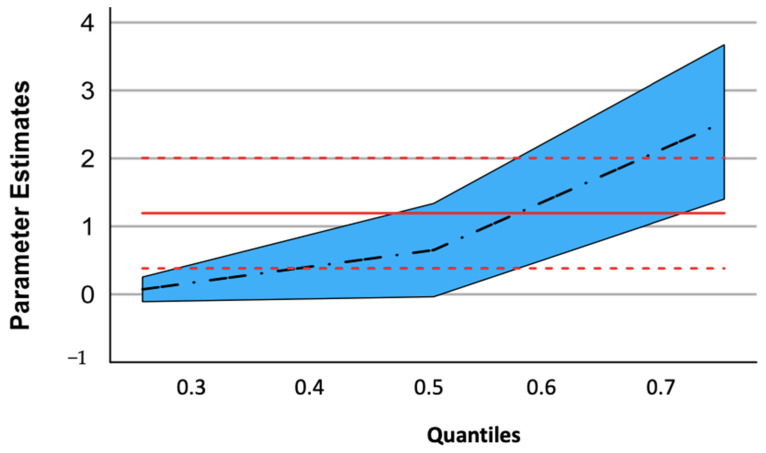
Quantile regression: the CRP gap between the MDD patients and the CTRLs by quartiles. The estimated parameter is the CRP gap between the MDD patients and CTRLs at the different regression quantiles. The blue area shows the confidence intervals of the estimated parameter, and the black dashed line indicates the estimated parameter at the different regression quantiles. The red line and dotted lines represent the ordinary linear regression.

After adjustment (age, sex, BMI, and smoking status), the quantile regression analysis showed a higher CRP for MDD patients from Q3 (*p* < 0.01) (Figure 2, Table 2). The threshold of Q3 corresponded to a CRP level of 2.15 mg/L. 

The logistic regression analysis showed an ODS ratio of 14.42 (95% CI, 2.53–81.9) *p* = 0.003 for the MDD patients versus the CTRLs from a CRP level of 2.15 mg/L (Q3) after adjustment for age, sex, BMI, and smoking status. The logistic regression analysis showed an ODS ratio of 18.27 (95% CI, 2.04–164.88), *p* = 0.01, after adjustment for a CRP level that ranged from 3 to 10 mg/L. 

### 3.2. Nutritional Data 

The MDD patient macronutrient data showed that 37.6% of the daily caloric intake comprised fat, with 14.3% representing saturated fatty acids (SFAs), 0.7% trans fatty acids (TFAs), and 8.7% monounsaturated fatty acids (MUFAs) (Table 3). Both the TFAs and SFAs were above the nutritional reference (NR) values but not higher than the CTRLs. The 8.7% MUFAs intake was below the NR and the CTRL values (*p* = 0.006). The MDD patient *n*-6 PUFA level was below the NR but did not differ from the CTRL value, and the *n*-3 PUFA intake was below both the NR and CTRL values. As a result, the MDD patient ratio of *n*-6 PUFA to *n*-3 PUFA showed a (superior) trend compared to that of the CTRLs. The total CHO intake was 44%, below the NR, with a daily dietary fiber (DF) intake that was below the NR and CTRL values. The free sugar (FS) intake of 22.6% was above the NR and CTRL values. 

**Figure 3:**
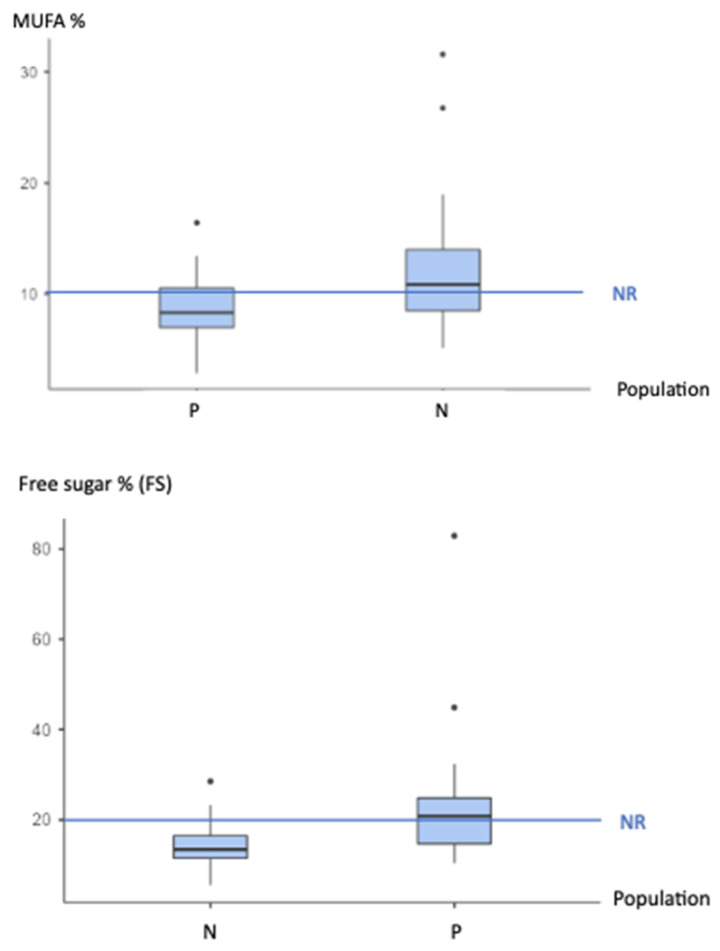
The higher nutritional risk factors for MDD patients. The data are presented as the mean ± the standard deviation of the mean. The nutritional references (NRs) are expressed as the percentage of the total daily energy expenditure, which corresponds to the daily theorical caloric need (101,102). %MUFA: the daily intake of MUFAs for MDD patients (mean = 8.756% ± 3.087) and CTRLs (mean = 12.144% ± 5.677) for an NF of more than 10%. % FS: the percentage free-sugar intake per day for MDD patients (mean = 22.6% ± 13.3) and CRTLs (mean = 14.40 ± 4.76) for an NR of 20%. The * singles points above the box plots could be statistically outliers, but clinically relevant.

For the FSs and MUFAs, the MDD patients showed higher risk factors than the CTRLs (Figure 3). 

Concerning the micronutrient and antioxidant data, the dietary intake of magnesium, zinc, and vitamins A and E among the female patients was below the NR and CTRL values (Table 4). For the male patients, the intake of all the micronutrients assessed was below the NR but not the CTRL values. The MDD patient intake of vitamin C was below the NR and CTRL values (Table 4). The MDD patient intake of fruits and vegetables (F&V), measured to estimate the dietary antioxidant intake, was below the NR and CTRL values. 

Overall, the dietary intake of protective nutrients for the MDD patients was lower than that of the CTRLs for vitamin C; F&V; and, depending on the sex, for magnesium, zinc, and vitamins A and E (Table 4). 

The food category data showed a daily intake of F&V, whole grains, and legumes below the NR for both populations (Table 5). The MDD patient daily intake of these food categories, except for whole grains (slight trend, *p* = 0.502), was below that of the CTRLs. 

The MDD patients’ AGE levels were more elevated than those of the CTRLs (*p* = 0.042). After adjustment (age, sex, BMI, and smoking status), the data did not remain significantly different (*p* = 0.343). 

### 3.3. Nutrition and Inflammation

In the univariate model, the CRP level of the MDD patients was positively associated with fat, SCFAs, TFAs, *n*-6 PUFA (trend), and FSs and negatively associated with the intake of F&V (Table 6).

**Figure 4:**
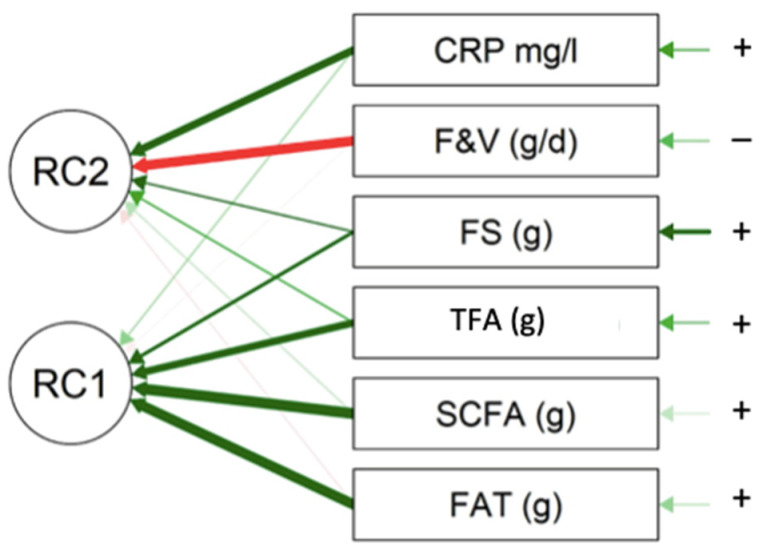
The PCA Varmix data, nutrients and F&V diagram. All the nutritional factors that correlated positively with the CRP level are represented with “+” and a green arrow. The food categories or nutrients that correlated negatively with CRP are represented with “−” a red arrow. The thicker the arrow, the more important the impact on the model. RC1 and 2 are the rotation axes that collate highly correlated variables together. This model was explained by two RCs.

The multivariate models showed that collectively, SCFAs, TFAs, fat, and free sugars (FSs) were positively correlated with the CRP level, in contrast to F&V (Figure 4) (Table 7). The impact of the pro-inflammatory nutrients in the model was shown via the uniqueness factor. In descending order, the impact was ranked as follows: SCFAs, fat, TFAs, and FSs. The PCA explained 48.9% of the model for PC1 and 69.7% for PC2. 

**Figure 5:**
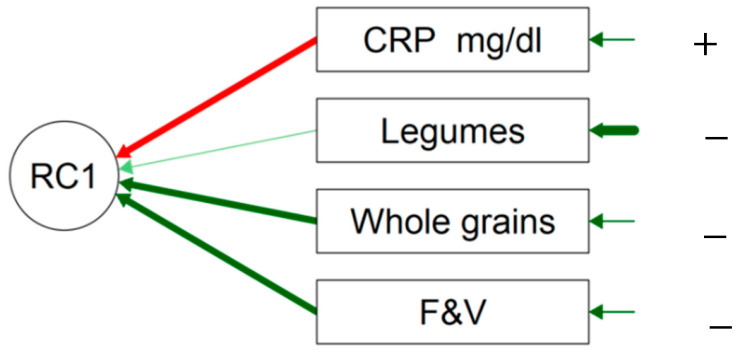
PCA/Varmix diagram of the food categories and the CRP levels for MDD patients. All the nutritional factors that correlated positively with the CRP level are represented with “+” and a red arrow. The food categories or nutrients that correlated negatively with the CRP level are represented with “−”and a green arrow The thicker the arrow, the more important the impact on the model. RC1 is the rotation axis that collates highly correlated variables together. This model was explained by one RC.

The protective food categories such as F&V, whole grains, and legumes were collectively and negatively associated with the CRP level (Table 8, Figure 5). The impact of whole grains and F&V was higher than that of legumes. 

## 4. Discussion 

### 4.1. CRP 

The MDD patients without comorbidities showed a CRP level higher than that of the CTRLs. The percentage of elevated CRP levels (above 1 mg/L) and LGI (above 3 mg/L) for the MDD patients was consistent with the literature [5]. After adjustment, the MDD patient CRP levels remained higher and fell above a threshold of 2.15 mg/L, with an odds ratio of 14.4 for the MDD patients compared with the CTRLs. The odds ratio for LGI range was 18.27. LGI is already identified as a cardiovascular risk factor. 

Thus, our MDD patients without pathological inflammation risk factors were associated with a higher CRP level “per se”, which was our research hypothesis. As we saw previously, inflammation could have an impact on mood disorders related to the sickness behavior syndrome. MDD is a chronic and serious illness whose severity criteria include a high prevalence of treatment resistance. Inflammation could also dampen the effectiveness of antidepressant drugs. Thus, inflammation could act in this context as a risk factor for depression symptoms; treatment resistance; and, therefore, the recovery of our patients. Certain studies have also shown that different types of antidepressants could be more effective when related to the level of CRP [24,25]. More studies are necessary to confirm this, but the inflammatory context could be an important risk factor to take into account in diagnosis and the choice of the treatment. 

### 4.2. Nutrients 

The MDD diet pattern was a high-fat (37.6%), high-sugar (22.6%) pattern with a deficiency in anti-inflammatory nutrients and a high glycemic load. This framework was consistent with the WD criteria. The control population showed a similar diet pattern, which was consistent with the definition of the WD and the national survey completed in Belgium [104]. 

In detail, both populations had an excess of pro-inflammatory fats such as TFAs and SFAs, with no differences between them. The intake of the pro-inflammatory fat *n*-6 PUFA was below the nutritional reference (NR) value for both groups, with no differences between them. Conversely, the intake of the anti-inflammatory fats MUFA and *n*-3 PUFA in MDD patients was lower than the NR and CTRL values. Thus, the MDD patients’ total fat intake was higher than the NR but lower than the CTRL values due to a deficiency in anti-inflammatory lipids. The MDD patients’ *n*-6-to-*n*-3 ratio showed a slightly superior trend due to the higher deficiency in *n*-3 PUFA, pushing it more towards the pro-inflammatory side. 

The amount of AOs evaluated by the F&V intake was lower for the MDD patients when compared to the NR and the CTRLs. The dietary intake of vitamin C was consistent with this result. Thus, the MDD patient diet was less protective against the oxidative stress and inflammation associated with depression [65]. Consistent with this result, we found a negative correlation between the CRP levels of the MDD patients and their F&V dietary intake. The magnesium, zinc, and vitamins A and E dietary intake was below the NR for both populations, but only the female MDD patients showed a lower intake than the CTRLs. These results could allow us to adjust nutritional strategies with more accuracy. Finally, the low dietary fiber intake of the MDD patients was a risk factor for the abundance and diversity of a healthy microbiota. It could weaken the integrity of the gut barrier and promote inflammation. 

Overall, the MDD patients showed aggravating nutritional factors when compared to the CTRLs, including more FSs, lower MUFAs, a higher trend in the *n*-6/*n*-3 ratio, and a higher deficiency in other anti-inflammatory nutrients such as DFs and AOs. 

### 4.3. Carbohydrates, Dietary Fibers, and Glycemic Load 

The carbohydrate (CHO) intake of the MDD patients was unbalanced at 44%, compared to 50% for the NR, whereas there was an excess of FSs and a deficiency in dietary fibers. The MDD patient F&V intake was below the NR and the CTRL values. A diet low in fruits and dietary fibers and at the same time rich in sugar is presumed to be rich in added sugar (e.g., desserts, snacks, sweet beverages, and ultra-processed food). The MDD patients’ dietary intake of whole grains was below the NR, with a slight trend below the CTRL values. Overall, these data were consistent with the high consumption of added sugar and refined cereals. Both of these food categories have a high GI and thus could lead to increased oxidative stress and could sustain an inflammatory context. Consistently, the diet of MDD patients showed a higher glycemic load frequency than that of the CTRLs. 

There is also a link between mood disorders and sugar. The consumption of sugar has an impact on the mood that occurs due to the release of neuropeptides in the reward pathway (RP), enhancing the mood [105,106]. Conversely, long-term sugar consumption could lead to neuronal changes in the RP. These changes could increase sugar sensitivity and mood disorders in a drug-like pattern [106]. 

From a metabolic point of view, a high-sugar, high-glycemic-load diet is a risk factor for insulin resistance, diabetes, and obesity [37]. MDD is a chronic mental disease. Thus, a long-term high-sugar, high-GL diet in the context of MDD could contribute to increasing the risk of developing diabetes and obesity in our patients [37]. Diabetes and obesity are frequent comorbidities of depression and are associated with inflammation “per se”. Thus, they could in turn sustain or reinforce an inflammatory context. 

### 4.4. Advanced Glycation End Products (AGEs) and the Western Diet 

An elevated AGE level has been associated with depression [43]. Thus, the AGE level of the MDD patients was presumed to be higher than that of the CTRLs. However, we did not find any difference between the populations after adjustment. This result could be explained by the WD intake of both populations. Moreover, smoking status was expected to increase the AGE level, and the heterogeneity of the smoker numbers within the two populations could have had an impact. 

### 4.5. Inflammation and Nutrition 

We found a positive correlation between the pro-inflammatory nutrients (SFAs, TFAs, *n*-6 PUFA, FSs, and fat) and the CRP level. Conversely, the negative association between the CRP level and the F&V dietary intake could be linked to their high content of anti-inflammatory nutrients, such as AOs, and fiber, as seen previously. 

In the multivariate analysis, the model consistently showed two areas: one group with CRP and the pro-inflammatory nutrients and a second one with the anti-inflammatory food category (F&V). In the univariate analysis, the Spearman coefficients were significant but not elevated for each nutrient, which contrasted with the multivariate analysis that explained 69% of the model. We could rely on these results with our hypothesis of the global action of the diet in relation to physiology. Food is a complex matrix and acts as a whole. We presumed that the balance of pro- and anti-inflammatory nutrients, as well as the microbiota, dietary fiber substrata, and glycemic load, could play a role in the inflammatory context. The food category multivariate analysis confirmed this hypothesis, whereas the main protective foods rich in anti-inflammatory nutrients, such as F&V, legumes, and whole grains, were negatively associated with the CRP level. 

Therefore, for the MDD patients, the ratio between risky and protective nutritional factors was unbalanced, leaning more towards the pro-inflammatory side and being less favorable than that of the CTRLs. 

### 4.6. Limits of the Study 

Our first limitation was the number of patients recruited. MDD inpatients without comorbidities are rare. These exclusion criteria were mandatory due to our research hypothesis concerning an elevated CRP level “per se”. Even if comorbidities were presumed to enhance an elevated CRP level, it would be necessary and interesting to evaluate the full spectrum of MDD patient comorbidities and determine if the same nutritional risk factors existed. For the nutritional data and the statistical calculation related to sex, a higher number of patients would be required for a confirmation, for men in particular. The second limitation was linked to the nature of our study. We performed a transversal study with a psychiatric trained protocol in the CRP assessment. The underlying hypothesis of the link between depression and nutrition was chronic inflammation. The chronicity could not be verified in this transversal study. However, our patients suffered from an acute and painful state of mind. Their vulnerabilities could make it difficult to perform blood tests on a regular basis.

For the nutritional calculations, we used the Nubel software database [96]. In the Nubel database, not all nutritional information was provided, and this applied to the micronutrients in particular. Furthermore, the database did not allow us to conduct accurate analyses on, for example, the differences between added and natural sugars and the intake of different types of *n*-3 PUFAs and TFAs. Nevertheless, this remains the reference database in Belgium that is used for national food surveys. 

## 5. Conclusions

The results of this study showed that MDD patients without comorbidities had an elevated CRP level when compared to CTRLs, and this particularly applied in the range of low-grade inflammation. The MDD patient diets had a global pro-inflammatory characteristic and showed more severe nutritional risk factors than the diets of the controls. We found that the excesses and deficiencies in nutrients, some of them related to sex, could contribute to this risky profile at the same time as a frequent high-glycemic-load diet. Therefore, this is the overall feature of the WD that could act as a risk factor. The association found between the CRP level and the nutritional risk factors emphasized the WD-related risk of sustaining an inflammatory context. The relationship between food and mood is complex. A high-sugar diet could enhance the mood, but in the long term it could contribute to a neural adaptation that could in turn reinforce sugar intake. Thus, MDD itself could enhance a pre-existing (or newly emerging) unbalanced diet. This vicious circle could sustain oxidative stress and inflammation in addition to comorbidities such as diabetes and obesity. Further studies are required to confirm these results, but this study highlights the importance of the WD’s global features and its management in the context of MDD. 

## Figures and Tables

**Table 1:** The descriptive data distribution of the MDD patient (P) and CTRL (N) CRP levels (mg/L). Note: N = number of patients and CTRLs.

Group	N	Mean	Median	SD	SE
0	32	2.39	1.80	1.97	0.348
1	31	1.13	0.850	0.871	0.156

**Table 2:** Quantile regression and the estimation of the parameters according to the different quantiles after adjustment: age, sex, BMI, and smoking status. The parameter is the gap between the CRP levels of the MDD patients and the CTRLs. Note: q = quantile.

Parameter	q = 0.25	q = 0.5	q = 0.75
Patient vs. CTRL	0.073	0.630	2.537
*p* value	0.345	0.065	<0.001
Confidence interval	−0.128/0.273	−0.042/1.301	1.401/3.673

Dep. Variable: CRP mg/L. Model (adjustment): age, BMI, population, sex, smoking status.

**Table 3:** Macronutrients: the MDD patients’ nutritional data compared to the NR and control values [100,101]. The data are presented as the mean ± the standard deviation of the mean. The nutritional references (NRs) are expressed as the percentage of the total daily energy expenditure, which corresponds to the daily theorical caloric need. For the dietary fibers, the NR is a daily intake (g). Statistical test legend: (^a^) Mann–Whitney test *p* value, (^b^) Student’s test *p* value, and (^c^) Wilcoxon test *p* value. A *p* < 0.05 was considered statically significant. A trend was considered a non-statistically significant *p* value (i.e., *p* ≥ 0.05) close to the 0.05 threshold.

Macronutrients/ NR	Patient	Gap vs. Nutritional References (NR)		CTRL	GAP vs. Nutritional References (NR)		GAP Patient vs. CTRL	
	Mean + SD	*p*-Value	No Difference (no)/Inferior (I)/Superior (S)	Mean + SD	*p*-Value	No Difference (no)/Inferior (I)/Superior (S)	*p*-Value	No Difference (no)/Inferior (I)/Superior (S)
**%FAT/** **<35%**	37.6 ± 4.83	0.003 ^b^	S	41.2 ± 5.77	<0.001 ^b^	S	0.009 ^b^	I
**%SCFA/** **<10%**	14.262 ± 3.845	<0.001 ^c^	S	14.830 ± 3.455	<0.001 ^b^	S	0.283 ^a^	no
**%TFA/** **0%**	0.709 ± 2.048	<0.001 ^c^	S	0.342 ± 0.182	<0.001 ^c^	S	0.989 ^a^	no
**%n-6 PUFA/** **4–8%**	2.127 ± 1.098	<0.001 ^c^	I	2.484 ± 1.343	<0.001 ^c^	I	0.360 ^a^	no
**%n-3 PUFA/** **1–2%**	0.330 ± 0.181	<0.001 ^c^	I	0.519 ± 0.338	<0.001 ^c^	I	0.017 ^a^	I
**PUFA n-6/n-3** **ratio**	7.441 ± 3.970			5.932 ± 4.035			0.051 ^a^	S (trend)
**%MUFA/** **>10%**	8.756 ± 3.087	0.015 ^b^	I	12.144 ± 5.677	0.961 ^c^	no	0.006 ^a^	I
**%Free Sugar (FS)** **<20%**	22.650 ± 13.314	0.673 ^c^	S	14.428 ± 4.760	<0.001 ^c^	no	<0.001 ^a^	S
**Fibres/** **25 g/day**	16.891 ± 7.102	< 0.001 ^b^	I	22.988 ± 6.397	0.048 ^c^	I	0.001 ^b^	I

Other data: % CHO intake = 44% ± 6.32 lower than the NR (50%) with *p* < 0.001. The gap between the daily caloric intake and the caloric theorical need (TDEE) was −124.01 kcal ± 549.68.

**Table 4:** Micronutrients. The MDD patient nutritional data compared to the nutritional references (NRs) and controls [100,101]. The data are presented as the mean ± the standard deviation of the mean. Statistical test legend: (^a^) Mann–Whitney test *p* value, (^b^) Student’s test *p* value, (^c^) Wilcoxon *p* value, and (^d^) Welch’s *p* value. A *p* < 0.05 was considered statically significant.

Micronutrients NR/Day (d)	Patient	Gap vs. Nutritional References (NR)		CTRL	GAP vs. Nutritional References (NR)		GAP Patient vs. CTRL	
	Mean + SD	*p*-Value	No Difference (no) /Inferior (I)/Superior (S)	Mean + SD	*p*-Value	No Difference (no)/Inferior(I) /Superior(S)	*p*-Value	No Difference (no) /Inferior(I) /Superior(S)
**Magnesium (mg)/d ** **M:350 mg ** **F:300 mg**	177.30 ± 72.38	M: <0.001 ^b^	I	186 ± 66.3	M: 0.003 ^b^	I	0.818 ^b^	no
	123.0 ± 58.3	F: <0.001 ^b^	I	198.0 ± 51.2	F: <0.001 ^b^	I	<0.001 ^b^	I
**Zinc (mg)/d ** **M:11 mg ** **F:8 mg**	5.00 ± 1.91	M: <0.001 ^b^	I	4.00 ± 0.33	M: <0.001 ^b^	I	0.460 ^d^	no
	2.67 ± 1.06	F: <0.001 ^b^	I	4.38 ± 1.4	F: <0.001 ^b^	I	<0.001 ^b^	I
**Vit A (µg)/d ** **M:750 mg ** **F:650 mg**	304 ± 187	M: <0.001 ^b^	I	384 ± 209	M: 0.009 ^b^	I	0.460 ^b^	no
	255 ± 152	F: <0.001 ^b^	I	513 ± 305	F: 0.007 ^c^	I	0.001 ^a^	I
**Vit E (mg)/d** **M:13 mg** **F:11 mg**	7.88 ± 5.41	M: 0.008 ^b^	I	5.95 ± 2.12	M: <0.001 ^b^	I	0.463 ^b^	no
	4.42 ± 2.60	F: <0.001 ^c^	I	6.95 ± 2.82	F: <0.001 ^b^	I	0.003 ^b^	I
**Vit C (mg)/d** **110 mg**	44.688 ± 36.866	<0.001 ^c^	I	74.1 ± 44.4	<0 .001 ^c^	I	0.001 ^a^	I
**AO /(F&V)** **550 g/d**	226 ± 182	<0.001 ^c^	I	451 ± 199	0.003 ^c^	I	<0.001 ^a^	I

**Table 5:** Food categories. The MDD patient nutritional data compared to the nutritional references (NRs) and controls. The data are presented as the mean ± the standard deviation of the mean (101,102). Statistical tests: (^a^) Mann–Whitney test *p* value, and (^c^) Wilcoxon test *p* value. A *p* < 0.05 was considered statically significant. A trend was considered a non-statistically significant *p* value (i.e., *p* ≥ 0.05) close to the 0.05 threshold.

Food Categories/ NR	Patient	Gap vs. Nutritional References (NR)		CTRL	GAP vs. Nutritional References (NR)		GAP Patient vs. CTRL	
	Mean + SD	*p*-Value	No Difference (no)/Inferior(I) /Superior(S)	Mean + SD	*p*-Value	No Difference (no)/Inferior(I) /Superior(S)	*p*-Value	No Difference (no)/Inferior(I) /Superior (S)
**Fruits & vegetables (F&V)** **550 g/day**	226 ± 182	<0.001 ^c^	I	451 ± 199	0.003 ^c^	I	<0.001 ^a^	I
**Wholes grains** **NR:125 g/day**	65.4 ± 63.8	<0.001 ^c^	I	68.0 ± 46.3	<0.001 ^c^	I	0.502 ^a^	I (trend)
**Legumes g/week** **NR:100 g/day**	32.8 ± 72.2	<0.001 ^c^	I	166 ± 207	0.831 ^c^	no	<0.001 ^a^	I

The MDD patients had a frequency of 59.1% ± 23.34 for highly glycemic meals. This frequency was higher than that of the CTRLs (41.8% ± 15.62), *p* = 0.001.

**Table 6:** Univariate analysis. The correlation between the CRP level and MDD patients was determined using the Spearman coefficient. *p* < 0.05 was considered statically significant. A trend was considered a non-statistically significant *p* value (i.e., *p* ≥ 0.05) close to the 0.05 threshold.

Nutrients	*p* Value	Spearman Correlation Correlation
Fat (g)	0.015	0.427
SCFA (g)	0.034	0.375
TFA (g)	0.006	0.476
PUFA n-6 (g)	0.07 (trend)	0.324
FreeSugar (FS) (g)	0.039	0.366
F&V (g)	0.022	−0.403

**Table 7:** The multivariate analysis data of the MDD patients. Component loadings: the uniqueness represents the variance that was unique to the variable and was not shared with other variables. Component characteristics: the cumulative value is the representativity of the model.

	PC1	PC2	Uniqueness
FAT (g)	0.910		0.168
SCFA (g)	0.943		0.097
TFA (g)	0.742		0.311
FS (g)	0.506	0.412	0.574
F&V (g/d)		−0.853	0.271
CRP mg/L		0.758	0.394

Component characteristics: cumulative PC1: 0.489/PC2: 0.697.

**Table 8:** PCA/Varmix multivariate analysis of the MDD patient food categories. Component loading: uniqueness represents the variance that was unique to the variable and was not shared with the others. Component characteristics: the cumulative value is the representativity of the model.

	PC1	Uniqueness
CRP mg/L	−0.685	0.531
legumes(g/w)	0.588	0.654
whole grains (g/d)	0.595	0.646
F&V (g/d)	0.746	0.444

Component characteristics: cumulative PC1: 0.431.

## Data Availability

The data presented in this study are available on reasonable request from the corresponding author.
